# Ethylene Glycol Poisoning Complicated by Cardiac Arrest and a Raised Lactate Gap: A Case Report

**DOI:** 10.7759/cureus.78743

**Published:** 2025-02-08

**Authors:** Dhruva Sharma, Roshan Sebastian

**Affiliations:** 1 Critical Care Medicine, James Cook University Hospital, NHS, Middlesbrough, GBR

**Keywords:** cardiac arrest, ethylene glycol, hemodialysis, lactate gap, self poisoning

## Abstract

Ethylene glycol, a common component in automotive antifreeze and various household and industrial products, poses significant health risks upon ingestion, whether accidental or intentional. Characterized by severe metabolic acidosis, calcium oxalate crystal formation, and diverse end-organ damage, ethylene glycol toxicity can be fatal, with a potentially lethal dose estimated at 1500 mg/kg. The parent compound is osmotically active, leading to the production of harmful metabolites, such as glycolic and oxalic acids, which contribute to metabolic acidosis, nephrotoxicity, and cardiac toxicity. Acute management strategies involve supportive care, administration of fomepizole as a competitive enzyme inhibitor, and renal elimination through dialysis. Furthermore, the lactate gap serves as an important diagnostic tool in ethylene glycol poisoning, highlighting discrepancies between measured and expected lactate levels, which can indicate metabolic acidosis and tissue hypoperfusion. We present a case of ethylene glycol poisoning complicated by cardiac arrest in spite of initiating treatment and the possible use of the lactate gap to predict severity.

## Introduction

Ethylene glycol is a colorless, volatile, and flammable toxic alcohol (C2H6O2) commonly found in antifreeze and some industrial solvents. Its sweet taste poses a significant danger if ingested, as it is rapidly absorbed through the gastrointestinal tract. Ingestion of ethylene glycol, either accidentally or in a suicide attempt, is characterized by severe acidosis, calcium oxalate crystal formation and deposition, and a wide variety of end-organ effects that may be fatal [1,2].

The potentially lethal dose of ethylene glycol is approximately 1500 mg/kg [1]. Elimination is first-order when the concentration is under 250 mg/dL, with a half-life of approximately 4-6 hours, and zero-order kinetics above 250 mg/dL, with a rate of 10 mg/kg. When alcohol dehydrogenase is inhibited, preventing metabolism, the elimination half-life is prolonged to 10 to 18 hours and is renally dependent [2].

The parent compound is osmotically active, with metabolites mainly consisting of glycolic acid, which is responsible for anion gap metabolic acidosis while oxalic acid is responsible for the associated end-organ injury and nephrotoxicity [1,2]. Acute management of ethylene glycol poisoning includes supportive care, an antidote (fomepizole or ethanol if fomepizole is not available, which is a competitive enzyme inhibitor), and elimination via dialysis [2].

The lactate gap in the context of ethylene glycol (EG) poisoning refers to the difference between measured lactate levels and the expected lactate levels based on the patient's clinical presentation. Understanding the lactate gap is crucial for the diagnosis and management of ethylene glycol toxicity, as elevated lactate levels can indicate metabolic acidosis and tissue hypoperfusion.

## Case presentation

A 54-year-old male presented to the Department of Emergency Medicine with a history of intentional overdose of antifreeze. He ingested about 800 mL of 45 w/v% with the intention to self-harm three and a half hours before presentation to the hospital. On examination, he was inebriated and hemodynamically stable with National Early Warning Score (NEWS) 4, pulse 83/min, blood pressure 131/95 mmHg, respiratory rate 13/min, temperature 36.6 degrees centigrade, and blood glucose 5.2 mmol/l. He was administered a fomepizole loading dose of 15 mg/kg with a total of 1000 mg in 500 ml of normal saline over 30 min in the Accident and Emergency Department, as per the discussion with the National Poisons Information Service (NPIS), and was advised to start hemodialysis, as the amount consumed was life-threatening.

His initial blood gas showed a high anion gap (19.6 mEq/L) with mild metabolic acidosis (pH 7.29) and an inappropriately high lactate level of 23 mmol/L on the blood gas machine. However, a repeat blood lactate test in biochemistry indicated a level of 2 mmol/L. His initial ethylene glycol level was 2833 mg/dl. He was transferred to the ICU and intubated for his airway protection and agitation. A percutaneous central venous line and hemodialysis catheter were secured, and he was started on hemodialysis with the intention of removing ethylene glycol metabolites. Hemodialysis was initiated with six hours of an initial loading dose of fomepizole. Continuous infusion of fomepizole with a dose of 1 mg/kg/hr was initiated.

The patient was on metaraminol support as a pressor after intubation and ventilation. Due to the risk of hemodynamic instability, continuous venovenous hemodialysis (CVVHD) was selected, using regional citrate for anticoagulation and calcium chloride infusion. The dialysis dose was set at 35 ml/kg/hr, with a blood flow of 130 ml/min and a dialysate flow of 2600 ml/hr. There was no ultrafiltrate, and the citrate and calcium chloride doses were titrated according to the protocol.

His ethylene glycol blood level, lactate gap, and blood pH improved on the filter as mentioned in Table 1 and Table 2.

**Table 1:** Ethylene glycol levels and other blood biochemistries Zero hours refers to the time of the patient's presentation to the Department of Emergency Medicine.

	Day 1 (3 hours)	Day 1 (5 hours)	Day 1 (19 hours)	Day2 (25 hours)	Day 3	Day4
Ethylene glycol level (mg/L)	2833	2560	1046	329	<50	<50
Ethanol level (mg/dL)	<100					
Bicarbonate (mEq/L)	16	18	21	20	24	27
Creatinine (micromoles/L)	79	89	61	72	34	64
Serum osmolarity (mOsm/kg)	368			305		

**Table 2:** Lactate level and blood pH initial 12 hrs Zero hours refers to the time of the patient's presentation to the Department of Emergency Medicine.

	0 hours	2 hours	4 hours	9 hours (cardiac arrest)	12 hours
Lactate Arterial Blood Gas (mmol/L)	23	16	7.3	6.6	4
Lactate Serum (mmol/L)	2.4	1.8	1.2	-	4.6
Blood pH	7.29	7.39	7.48	7.35	7.37
Ionized Calcium (mmol/L)	1.25	1.20	1.13	1.15	1.08

After almost nine hours of treatment in the hospital, the patient experienced a brief episode of cardiac arrest with pulseless electrical activity lasting eight minutes before the return of spontaneous circulation. Pre-arrest values were pH 7.35, potassium 3.5 mEq/L, and ionized calcium 1.15 mmol/L.

Post-cardiac arrest, the patient required infusions of noradrenaline, vasopressin, and adrenaline to maintain blood pressure, and CVVHD resumed gradually. A bedside echocardiogram indicated biventricular impairment, with a fixed dilated inferior vena cava (IVC) and reduced cardiac output, showing a left ventricular outflow tract (LVOT) velocity time integral (VTI) of 9 cm.

The fomepizole protocol and hemodialysis continued until the ethylene glycol level was undetectable, with levels monitored serially. The patient was taken off dialysis on day 3.

While in the ICU, he developed hospital-acquired pneumonia and was started on IV piperacillin and tazobactam. His CT brain findings were non-significant and he was successfully extubated on day 8 of his admission.

## Discussion

Toxicity from ethylene glycol is mainly related to its metabolites, glycolate and oxalate [3]. Ethylene glycol-related mortality significantly increases if glycolate concentration exceeds 12 mmol/L [4,5]. The spectrum of organ dysfunction includes neurotoxicity, cardiopulmonary involvement, and acute kidney injury, mainly related to oxalate [2]. The clinical progression of ethylene glycol poisoning includes a neurological stage occurring from the initial 30 minutes to 12 hours, followed by a cardiopulmonary stage lasting from 12 to 24 hours, and lastly, a renal injury stage from 24 to 72 hours, causing acute kidney injury [6-8].

The cardiopulmonary symptom spectrum comprises tachycardia, hypertension, hyperventilation, congestive heart failure, arrhythmia, and acute respiratory distress syndrome [7,8]. Cardiac arrest in ethylene glycol poisoning is rarely reported. It is possibly due to the direct toxic effect of the drug causing myocarditis when treatment is not initiated or is delayed [9]. In our case, the patient experienced cardiac arrest after the initiation of hemodialysis and fomepizole, without any acidosis, and showed reduced cardiac function on echocardiography done post-arrest, making this situation unique and worthy of further research.

Possible causes for the cardiac arrest include the direct cardiotoxicity of metabolites, hemodynamic instability, and hypocalcemia, especially when the patient is on citrate regional dialysis. Additionally, a poorly positioned hemodialysis catheter can lead to ineffective dialysis, delaying the clearance of toxic metabolites.

Early prevention of systemic complications involves the administration of an antidote to prevent further toxic metabolite formation and to reduce calcium oxalate precipitation in the kidneys. The NIPS protocol recommends an adult loading dose of fomepizole at 15 mg/kg IV over 30 minutes, followed by maintenance doses of 10 mg/kg IV every 12 hours for four doses, after which the dose should be increased to 15 mg/kg IV every 12 hours. If renal replacement therapy is initiated more than six hours after the last fomepizole dose or if no fomepizole has been given, a loading dose of 15 mg/kg IV over 30 minutes should be administered, followed by a continuous infusion of 1 mg/kg/hr or 10 mg/kg IV every four hours. The dosing for pregnant women and children is weight-based. In cases of unavailability or delay, ethanol should be administered urgently, followed by fomepizole when available, with caution for patients with depressed consciousness levels, pregnancy, children, and hepatic disease.

An effective way of removing metabolites from the system is extracorporeal treatment (ECTR) or hemodialysis, as the antidote delays total effective metabolism in the hope that ethylene glycol pharmacokinetics remain within a non-toxic acceptable range [10]. The debate about early vs. late ECTR is explained with EXTRIP workgroup recommendations, but nothing is mentioned about preventing cardiac arrest. Uncertainty remains regarding the mortality benefit of ECTR vs. non-ECTR management and patient outcomes at different plasma levels of glycolate. Another major issue with EXTRIP recommendations is the problems encountered in various institutions regarding the turnaround time for glycolate and ethylene glycol levels, which complicates the decision to start ECTR [4].

The lactate gap in ethylene glycol poisoning is commonly affected due to additional unmeasured anion glycolate [8]. Glycolic acid is structurally similar to lactate and can use lactate oxidase to produce hydrogen peroxide, causing falsely elevated lactate levels in machines using this method, necessitating further evaluation for use as a bedside surrogate marker for glycolate levels [11,12]. The lactate gap is calculated as the difference between the measured lactate concentration and the expected lactate concentration based on the clinical context.

High concentrations of ethylene glycol are not an indication of ECTR, as it is the metabolites that are toxic and cause organ dysfunction.

The EXTRIP workgroup mentioned various indications for ECTR based on ethylene glycol level, osmolar gap, and anion gap. Additionally, coma, seizures, and kidney impairment are recommendations for ECTR, although with low-quality evidence [4].

## Conclusions

Cardiac arrest related to severe acidosis in ethylene glycol poisoning has been reported in various case reports. In this patient, cardiac arrest occurred after the initiation of the antidote and hemodialysis, with mild acidosis. This may be due to the direct toxic effect of ethylene glycol and its metabolites, indicating the need for early initiation of hemodialysis despite antidote therapy when high concentrations of ethylene glycol are consumed.

Secondly, to monitor the toxicity of ethylene glycol, glycolate levels are not available in every hospital. One can use the lactate gap as a surrogate marker for it, as further research is required to establish a correlation, given that lactate is a bedside investigation that can help guide therapy in ethylene glycol poisoning. Elevated lactate gaps have been associated with more severe toxicity and higher mortality rates, highlighting the potential utility of the lactate gap in risk stratification.
